# Hospital and Institutional News

**Published:** 1911-11-04

**Authors:** 


### HOSPITAL AND INSTITUTIONAL NEWS.

### A RECORD OF PERSONAL SERVICE.

The world of philanthropy is to-day the poorer by the death of Mr. Charles Bowles Hare. Such a man can ill be spared, and in Bristol and the West his loss seems irreparable. A fascinating personality, he created an impression that was ineradicable. Slim and active, his dark hair and military manner were in curious contradiction to his three score years and ten. The key-note of his life was Energy; and there was no branch of Bristol life—social, political, municipal, patriotic, musical, or (more than all) charitable—in which his hand was not either at the helm or prominently and actively concerned. Mr. Hare was one of the "merchant princes" of Bristol. The sole owner of one of the great Bristol businesses, founded more than a century ago by his grandfather, Mr. John Hare, he was to the last actively concerned in its welfare. The great colour works, stretching for many acres by the banks of the Avon, must be familiar to all entering Bristol by rail from the south-west. It would seem more than enough for one man to handle such vast interests. But we find him on the directorate of the Bristol Waterworks, Bristol Suspension Bridge Co., and Bristol Brewery. As a committee-man his calls were innumerable. He was a member of the Committees of the Bristol General Hospital, Clifton Royal Convalescent Home, Hooks Mills Asylum, Weston Convalescent Home, Bristol Docks Committee, Bristol Art Gallery, and Bristol Musical Festival. And let it be understood that he was no lay figure. That indomitable energy which put to shame the feeble efforts of men half his age would never allow him the *dolce far niente* with which so many committee-men are afflicted. Once let his name be associated with a project and its success was assured. He was forty years a Town Councillor, thirty years a magistrate, Master of the Society of Merchant Venturers in 1876, Sheriff of Bristol in 1878, a past president of the Colston Parent Society, and ex-churchwarden of St. Mary Redcliffe Church (pronounced by Queen Elizabeth to be "the fairest and goodliest church in her realms"). But among all his countless interests hospital work in general, and that of the Bristol General Hospital in particular, lay nearest his heart. It may be confidently asserted that he took more pride in being a vice-president of the "B.G.H." than in any of the other honours that crowded on him. It is 28 years ago since he first took his seat on the Committee, and what he has been to the institution during that time can never be estimated. That genius for finance which had made him a very wealthy man in his business was unreservedly poured out for the good of the Hospital. No detail was too small and no scheme too great for him. Only this year the Committee have appealed to the public for £45,000 for an extension which was urgent and essential. The amount is now nearly raised, and mainly through his efforts. His knowledge of hospital management was unrivalled. Some years ago he inaugurated a system of accounts by which waste was reduced to a minimum, and much money has been saved. He has for nearly thirty years been foremost in keeping his Hospital abreast and even ahead of the times, and the honourable position it to-day occupies is largely due to him. His health of late had not been good. A severe attack of epistaxis in 1909 had made his friends anxious for him. But with a courage that was characteristic he disdained the slothful retirement that seemed indicated and worked to the last. His tragic and sudden death at the Bristol Exhibition cast a gloom over the whole city. Bristol can find no one to adequately fill his place. At the Clifton and Constitutional Club, at the Commercial Rooms, on Municipal Committees, most of all at the Bristol General Hospital—he will be sadly missed. But he had earned his rest. His was a life of work. He died—as he would have wished—in harness.

### THE GENERAL MEDICAL COUNCIL ELECTIONS.

The registered practitioners of England and Wales are to elect this month three members to the General Medical Council. As several of the candidates are or have been closely connected with hospital matters in various districts, the result of this election will be of considerable interest to the institutional world, though probably there is no vital principle at stake which will be affected one way or the other according to the success or failure of any particular candidate to secure the suffrages of his fellow-practitioners. Dr. Langley Browne, of West Bromwich, and Dr. Latimer, of Swansea, are retiring members of the Council who seek re-election. The former is consulting surgeon to the West Bromwich Hospital, and has written upon the statistics of ten years' surgery at that institution, as well as upon many strictly technical subjects. The latter is consulting surgeon to the Swansea Hospital and has been medical officer to the Swansea Dispensary; he is surgeon to two railway companies, and has contributed both to The Hospital and to other papers; he has taken also especial interest in industrial diseases, such as chest complaints among copper workers. The other candidates include Mr. Geo. Jackson, of Plymouth, who has previously served on the General Medical Council; he is honorary surgeon to the Devon and Cornwall Ear and Throat Hospital, the Plymouth Public Dispensary, and the Deaf Mute School, and consulting surgeon to the Provident Dispensary. Mr. Geo. Brown, of Callington, Cornwall, is editor of the *Medical Times and Hospital Gazette*. Dr. S. C. Lawrence, of Lower Edmonton, is Medical Officer of Health for that urban district, and is the only London candidate. He has held numerous hospital appointments, in the Colonies and at home, chiefly in the Midlands. Dr. A. Hawkyard, of Leeds, is a barrister as well as a doctor. Mr. T. J. Verrall, of Bath, was formerly in practice at Brighton, where he was surgeon to the Sussex County Hospital; the Brighton and Hove Lying-in Institute and Hospital for Women, and the Brighton Provident Dispensary.

### THE MIDDLE-CLASS PATIENT.

In the address delivered by Sir James Crichton-Browne, printed in our last issue, emphasis is laid upon the oft-told tale of the advantages to the poor of receiving in voluntary hospitals the gratuitous services of the very men to whom the wealthy have to pay large fees. Sir James narrated as an instance of this the story of two girls who recently underwent the same operation. One was the child of rich parents, who paid between two and three hundred guineas altogether to the medical men in charge of the case. The other was of poor parents, and received exactly the same attention and skill in a public hospital; her friends paid for this not a single penny. This story is not only true, but quite a common one; such things happen every day, in scores and hundreds of cases. Where, however, the system contains a serious omission is in its failure to provide for the middle-class patient, who can neither pay these large fees for consulting physicians and surgeons, nor be admitted to a hospital as one suitable for charitable relief. There are, of course, a certain number of agencies whereby skilled attention and accommodation in a nursing home can be secured at moderate cost for those who are not of the hospital class; such as St. Thomas's Home, the Nightingale Home in Lisson Grove, and St. Saviour's Hospital, to quote a few London instances. And the medical profession—general practitioners and consultants alike—do an amount of work either quite gratuitously or for nominal fees for this class of patient which is almost unsuspected and quite unknown. Notwithstanding this, it is perfectly accurate to say that the rich and the poor enjoy a degree of care and attention during serious illness which the middle class, the real backbone of the nation, cannot as a rule obtain. This fact has long been admitted, and has long been overdue for reform. Such reform might well engage the attention of those who are busy with schemes for the amelioration of the lot of those whom sickness overtakes. The National Insurance Bill will not affect this class, except in so far as it will tax them even more heavily than at present. Yet we are convinced that some provision for those who can and do pay their doctor moderate fees for ordinary attendances, but cannot face the tremendous expenses of long illnesses, operations, nursing homes, consultations, and so on, would be of more lasting benefit to the State and its citizens than many of the provisions of this measure as at present constituted.

### CHINESE DECORATIONS FOR PLAGUE DUTY.

Whether the present Government of the Chinese Empire maintains its authority or not seems at the moment a difficult question to decide. But at least it is pleasant to see evidence that the Emperor's advisers are alive to the value and importance of work done in combating the devastations of pneumonic plague, which has been epidemic in certain parts of the Empire. That such is the case may be inferred from the list of decorations, licences to wear which are published in the *London Gazette* for October 27. The following have authority from the King to accept and wear the First Class, Third Division, of the Imperial Chinese Order of the Double Dragon: William H. G. Aspland, M.D., Professor at the Union Medical College, Pekin; J. M. Stenhouse, M.B., of the same College; B. S. Browne, M.B., of the Church Missionary Society Hospital, Ningpo. Similarly gazetted to the Third Class, Third Grade, of the same Order, is Mr. G. G. Wilson, Secretary and Treasurer of the Pekin College above mentioned.

### THE ATTACK ON GUY'S HOSPITAL.

The Southwark Guardians have obtained a little cheap notoriety by denouncing Guy's Hospital and its management as the very acme of abomination. The public, we have reason to know, is astonished and alarmed, and it may be well to turn the limelight of fact upon the Guardians of Southwark and their policy of neglect which has been for years the main cause of nearly all the trouble. The infirmary and hospital accommodation provided for the Guardians is situated at Dulwich, some miles from the borough, and the Southwark Guardians have not provided, as they ought to have provided long ago, a receiving house with a number of beds and proper medical and nursing provision, where accident and other cases of emergency which properly belong to them as Guardians of the Poor can be received and treated. No voluntary hospital can do the work of the Poor Law Guardians without sanctioning abuse and committing an injury upon the really deserving poor whom it was established to protect. Let the Southwark Guardians purge their contempt first by doing their duty towards the poor of Southwark which they have too long neglected. We hope the Local Government Board will hold a public inquiry into this matter without delay and so bring the Southwark Guardians to a sense of their plain duties and responsibilities.

### THE CASE OF MRS. MALLANDAIN AND GUY'S.

The authorities of Guy's Hospital have not thought well so far to reply to the statements appearing in the Press in connection with this case. Mr. William Mallandain, of 131 Tooley Street, has published the following statement of what actually occurred: "The death of my wife has been the cause of a lot of correspondence in the papers, she falling out of a window twenty-two feet from the ground. She was taken to Guy's at 3.30 A.M. and had to wait an hour and a half for a doctor with a fractured femur and badly lacerated leg, from which she lost a lot of blood, this being caused by falling on the glass of the shop lamp. All that was done by the nurse until the arrival of the doctor was to put a hot-water bottle to her feet and side. When the doctor arrived he covered the laceration with lint and bandage and asked the policeman if he could take her on the ambulance to the Rotherhithe Infirmary. I explained I was a tradesman and asked if he could telephone to another hospital. This he did for me, and she left Guy's at 5.30 A.M. for the London Hospital. Before leaving, the hot-water bottles were taken away by the nurse, although my daughter asked if they might remain, as her mother was in night attire, promising to return them as soon as she came back from the London Hospital, but the nurse said she might want them for another case, so my wife had to go about two miles through the cold streets in the early hours of the morning without them, although so large a hospital must have more than two bottles. I believe if she had been attended to more promptly at Guy's and not sent that long distance (every jolt of the ambulance over the rough stones causing her pain) her life might have been saved." From the above it would appear that it was an accident case of severe compound fracture; that it was taken to the hospital at 3.30 A.M. and, following the modern hospital practice, it would have been placed at once in one of the dozen or so emergency beds kept vacant for the reception of such cases. This system of reserving a limited number of beds for such cases does not appear to have prevailed at Guy's Hospital in the past, but we should hope that the authorities will now see their way to fall into line at once with the general practice. By so doing they will remove the risk of much avoidable suffering to many injured people, satisfy the just requirements of public opinion, and put an end, if the Local Government Board make prompt inquiry into the conduct of the Southwark Guardians, to the so-called scandals at Guy's Hospital which have of late frequently appeared in the Press, to the disadvantage of a great and admirable hospital of supreme importance to the suffering poor and the profession.

### CHILD MOTHERS.

At a meeting held at Fulham Palace in connection with the Fulham Ladies' Rescue and Preventive Association, the Bishop of London said the Association had to deal with those cases of girls under fourteen years of age. How to deal with such matters in the young is a very delicate and difficult problem. The most ancient known Babylonian laws may be said to be based upon sounder knowledge of physiology than the Hebraic code in relation to breaches of morality between the sexes. Death for both sexes was the penalty in the case of the married or the betrothed, but where the girl was unbetrothed only the man or youth who had deprived her of her honour was put to death. Against the girls referred to by the Bishop, boys of sixteen and seventeen years are said to have been the offenders. It is not suggested that Babylonian methods of punishment should be revived, but a lesson may be learnt from their attitude towards offences in the matter. Instances are rare in which physiological impulses are so developed in unmarried girls that they readily run the risk of bringing upon themselves life-long unhappiness. Not only is this so, but even in these days girls apparently can occasionally plead and plead truthfully—what probably Babylonian girls could not plead—innocence. Teaching upon these subjects is apt to be avoided. Possibly in the case of the girl, teaching which impressed certain physiological truths might do good. As regards the boys, similar knowledge and an appeal to a sense of manly honour that it should be one of their first duties to be a girl's guardian in such an important matter, might not be without effect. There are, however, no doubt other influences at work which tend to produce unhappy results in the relations between the sexes in the young. Overcrowding, especially when associated with drunkenness and immorality in parents, may lead to a disregard of all conscience as to the present or sense of responsibility with regard to the future in the matter.

### SPRUE.

The deadly effects upon human life and health in the tropics of the widespread tropical disease which is known as sprue are not comparable with those of malaria, yellow fever, sleeping sickness, and some others; but they are quite serious enough, and it is therefore satisfactory to learn that there is a prospect of an attack upon the problem of the ætiology of this disease. The solution of this question will obviously place in the hands of the tropical hygienist a powerful weapon of defence, and may very likely lead to an efficient system of prophylaxis. The Ceylon Government has offered the London School of Tropical Medicine the sum of £750 for the investigation of this disease, and according to the daily Press there is a prospect of a further contribution of £250 for the same research from the Ceylon Tea Planters' Association. It should not be beyond the bounds of possibility to secure the augmentation of this substantial nucleus through the private benefactions of persons whose wealth is derived from the districts where sprue is endemic. Ceylon, the Straits Settlements, Indo-China, Java, Manila, and Japan are all subject to the visitations of this disease, and cases are sometimes reported from the West Indies. The characteristics of the disease point with almost absolute certainty to a bacterial origin, and it is sincerely to be hoped that before a well-organised and well-equipped campaign the secrets of sprue will be disclosed, and that this condition will be added to the list of those tropical curses which modern research is steadily ameliorating and gradually annihilating.

### HOSPITAL VILLA FOR BEXLEY ASYLUM.

At a meeting of the London County Council on October 17, the Asylums Committee reported that considerable difficulty was experienced at Bexley Asylum in the treatment of male patients suffering from dysentery and phthisis largely by reason of the lack of facilities for supervision. The phthisical and dysenteric patients were housed at present in the east villa, which was built specially for chronic cases, and was quite unsuitable for its present occupants; moreover, there was no provision for open-air treatment. The committee therefore proposed that a hospital villa for the accommodation of thirty patients should be erected, and that the east villa should be used, as was originally intended, for the accommodation of the patients who work on the farm and in the gardens. It was decided to approve the expenditure of £60 in respect of the preparation of plans and an estimate of the cost of such a hospital villa.

### WORCESTER OPHTHALMIC HOSPITAL.

At a recent meeting of the Committee a letter was read from Dr. Pollard, stating that owing to the increasing calls upon his time he was reluctantly obliged to tender his resignation. Dr. Pollard has been on the staff of the Ophthalmic Hospital for nearly twelve years, and his resignation was accepted with great regret. A vote of thanks for the valuable work he has done for the hospital and patients during the time he has been connected with the institution was proposed by the chairman and unanimously passed to him. It was decided to advertise for an honorary surgeon and to make the appointment in December. A communication was read at the same meeting from the War Office, asking for the co-operation of the authorities in providing trained nurses in time of war. Compensation would be paid for the inconvenience incurred. The chairman was requested to write expressing the Committee's desire to assist in the proposed scheme.

### ACCOMMODATION FOR IMBECILES.

The need for additional accommodation for imbeciles under the Metropolitan Asylums Board came up for discussion at a meeting of the Managers on Saturday last, and it was decided to request the Local Government Board to sanction the use of the Fountain Hospital as a temporary asylum for the reception of unimprovable imbeciles. This hospital was detached from the fever service in January last and allocated for the accommodation of feeble-minded girls under the control of the Children's Committee, and would give room for the 500 unimprovable inmates to be transferred from Darenth. Some few years ago the buildings known as the Belmont Schools at Sutton were used for a similar purpose. The question is now becoming an urgent one, and, pending the consideration of a larger scheme for the future, the Managers are anxious to adopt any suitable temporary measure which could be carried out without delay, and which would relieve Darenth Asylum on a sufficiently large scale to enable effect to be given to the comprehensive scheme already approved for dealing with the imbeciles and feeble-minded.

### THE ENGLISH HOSPITAL AT NICE.

This month the Queen Victoria Memorial Hospital at Nice will be reopened. Considerable alterations have been carried out in this institution since last season, the most important of which is the provision of quarters for a resident medical officer. It has only recently been decided by the Committee to add a resident medical man to the staff, which has hitherto been entirely an honorary one. The successful candidate is Dr. Lang Todd, M.B., who has held, among other appointments, that of resident medical officer at the French Hospital in London. The plan initiated at the Queen Victoria Hospital last year of supplying nurses for private work has acted admirably, and has proved of great value to the English and American colony on the Riviera. This institution is the only one at Nice doing this work, and the Committee has made such complete arrangements for the ensuing season that visitors can rely on obtaining the services of trained nurses from England.

### THE ST. KATHERINE'S "HOSPITAL" FUND.

At the first meeting of the London County Council since the vacation on October 17, Mr. W. C. Johnson asked the chairman of the Local Government Committee (Mr. Geoffrey Drage) whether his attention had been called to certain articles concerning St. Katherine's Hospital in which it was stated that the site of this foundation, in one of the poorest districts in London, was in 1825 purchased by the Dock Company for over £160,000; whether one of its objects, "the relief of sick in the neighbourhood," had been entirely neglected since then, and a home established by Regent's Park for "Brothers and Sisters"; whether the brothers were clergymen who were allowed £400 a year apiece, together with attractive quarters; and whether the Local Government Committee would inquire "into the circumstances connected with this cruel misappropriation of money from Whitechapel and report thereon to the Council." In reply, Mr. Geoffrey Drage said that his attention had been called to the articles, and the matter would be inquired into by the Local Government Committee. Mr. W. Johnson is one of the representatives of Whitechapel on the Council, and there seems to be unfortunately some grounds for the use of the term "cruel misappropriation" of funds originally intended for the assistance of the sick and distressed in Whitechapel. A thorough investigation of the whole question would be opportune at the present moment, for we understand that the "Master," who received £800 a year and a fine house for duties which were apparently sinecure, has recently died, and no successor has as yet been appointed. If indifference or dilatoriness allows that to take place before the matter has been sifted and practical reform effected, the chance of any change which will restore their patrimony to the poor of Whitechapel may be delayed very considerably.

### THE RESPONSIBILITY OF HOSPITAL CHAPLAINS.

We are glad to see that an important place at the quarterly meeting of the Leicester Infirmary was given up to an appeal to ministers of religion to uphold the claim of Hospital Sunday. We have often had to point out that Hospital Sunday, owing to some unexplained weariness on the part of the clergy of all denominations, has not maintained its old tradition or produced its old amounts. Quarterly meetings take an added interest and value when they include, beside the routine work, important reminders to the world outside of its responsibility towards our voluntary hospitals. Hospital Sunday is typical of all the voluntary support of hospitals, and if it decreases in popularity ministers of religion must be held to blame. Hospital chaplains have a peculiar responsibility in this matter. Their work lies quite as imperatively in the pulpits of their own churches as in the wards of the institution to which they are attached. The Rev. J. T.

Coward, the Leicester Infirmary chaplain, has set a stirring example on Hospital Sunday there. Let us hope that his action will be the leaven which will leaven the whole lump. His individual effort should inspire others. To-day at any rate we put this question to his fellow chaplains: What answer could you make if at the next annual meeting you were asked by the institutional board and staff, "What have *you* done during the year for our hospital?" Not all hospital chaplains we fear could answer this as cheerfully as Mr. Coward. The chaplain, like the secretary, should be made to give an account of his stewardship.

### PHYSICIAL TRAINING.

An interesting memorandum has been issued by the Board of Education to the Secondary Schools in Wales on Physical Training. Stress is laid upon the opinion, now so widely held, that physical training aids in the development of the mind, not only indirectly by improving the health of the body, but directly. It is mentioned that the term "Physical Training" is used in a wide sense. The meaning is not limited to physical drill or gymnastic exercises but includes also games. Games and sports, however, tend to produce a somewhat unequal development of the body and require the corrective effect of physical exercises. The severest system is advocated. The present condition of most of our elementary schools is not favourable to the adoption of the Swedish system in its entirety owing to the fact that some physical apparatus is required. In secondary schools, however, the memorandum recommends that a gymnasium should be fitted out. It may be of practical interest to mention some of the recommendations with regard to the gymnasium itself. Amongst others as to size, character of ceiling, and of other not unimportant features it is stated "if practicable there should be a continuous line of windows on each side, and these should be made to swing open as centre-hung casements in order to provide ample light and effective cross-ventilation. There should be a blank wall-space of 9 feet up to the under-side of the sills in order to provide space for fixing the necessary apparatus. At least one end-wall should be left without windows, in order to provide a blank wall towards which the pupils can face in jumping, etc., and thus avoid liability to accidents likely to be caused by dazzling light. The floor should be constructed of narrow boards laid on wooden joints; wood block floors laid on concrete are unsuitable as they are inelastic. The gymnasium should be provided with means for warming in winter, as satisfactory work cannot be done in an unduly cold room." It is further mentioned that whatever room is used it must be a room which "can be easily cleaned and swept in every part" and that the lighting must be good. "A badly lighted room excites a depressing effect and thus deprives the lesson of some of its value."

### THE ROYAL FREE AND WOMEN STUDENTS.

The Earl of Sandwich presided on October 24 at the Quarterly Meeting of the Committee of Management at the Royal Free Hospital, Gray's Inn Road. The Report of the weekly board for the past quarter was adopted on the resolution proposed by Mr. Chaplin, who alluded to the fact that an extension of the buildings for the provision of additional departments was becoming an urgent necessity; and that further financial support from annual subscriptions and donations was greatly needed. A new agreement has been entered into with the London (R.F.H.) School of Medicine for Women, which provides *inter alia* for the annual payment of £100 to the Hospital for the facilities given to the students of the School. Lord Sandwich wishes it to be more widely known that gifts in kind to the Hospital would be most welcome, particularly fruit, vegetables and game, which would materially decrease the cost of provisions. This desire is one that must be shared by many other hospital presidents, though hospitals cannot exist on gifts of game alone.

### PROPOSED POOR LAW HOSPITAL FOR CHILDREN IN MANCHESTER.

A joint conference of Poor Law guardians has been held in Manchester for the purpose of considering a proposal to erect a children's hospital for the three unions of Manchester, South Manchester, and Prestwich. For some time overcrowding has obtained in the children's hospital wards at Withington and Crumpsall, and the South Manchester Board is already pledged to extensions. The Local Government Board has suggested that joint action is called for, and representatives of the three unions were met by Mr. Lowry and Dr. Fuller, of the L.G.B. No definite decision was arrived at, though the conference seemed favourably disposed to the idea in view. Should action in this direction be deemed feasible, the South Manchester Board would, of course, stay its hand so far as Withington extensions are concerned.

### CHELTENHAM GENERAL HOSPITAL.

At a special meeting of the Board of Governors of the Cheltenham General Hospital, held on Tuesday, October 24, a vacancy on the honorary surgical staff of the hospital, caused by the resignation through ill-health, of Mr. Herbert Bramwell, was filled by the appointment of Mr. J. C. R. Braine-Hartnell, of Weston House, Cheltenham. Mr. Braine-Hartnell, whose qualifications are F.R.C.S. Edin., M.R.C.S., and L.R.C.P. Lond., received his medical education at the Middlesex Hospital, London, where he obtained the Senior Broderip Scholarship. His present appointments include the Medical Officership to the Cheltenham Training College and Obstetric Surgeon to the Cheltenham District Nursing Association. At the same meeting Dr. S. M. Hebblethwaite, of Mangalore, Cheltenham, was appointed Honorary Clinical Pathologist to the Institution in the place of Dr. McLeod Munro, who had resigned the post preparatory to leaving the town. Dr. Hebblethwaite, who is an M.D. of the University of London, has been in actual practice for twenty-five years, and was for three years Resident Surgeon to the Worksop Dispensary and Medical Officer of Health for the same town for the last two and a half years of his residence there. He has also been engaged in practice in Yorkshire, and was for sixteen years

Medical Officer of the Harewood District of the Wetherby Union and Public Vaccinator for two districts.

### THE ROYAL ORTHOPÆDIC HOSPITAL, BIRMINGHAM.

This useful charity, one of the oldest in Birmingham, has just held its ninety-fourth annual meeting. The demands on its resources have shown no signs of diminishing, and, as the chairman, Mr. James Keay, pointed out, the Royal Orthopædic and Spinal Hospital is the only one in the city which devotes a large proportion of its income to the supply of instruments and apparatus for the relief of deformities. Inasmuch as other Birmingham hospitals supplied instruments in the past and have now ceased to do so, this hospital has to meet a correspondingly greater demand. The accounts for the year show a deficit of £962, and but for extra donations, the loss during the past year would have been more like £1,200. The most pressing reform is that of the rebuilding of the institution. Although up-to-date as regards the means for surgical treatment, the in-patients are housed in a building which is by no means adapted to the purpose for which it exists. The committee has to face this coming year an additional call for increased rent for the out-patient department, and difficulties are apprehended in connection with the effect of the Insurance Bill, so that it is anticipated that a special appeal for help will probably be necessary.

### LEICESTER'S NEW TUBERCULOSIS DISPENSARY

Following the example of many public health authorities, the corporation of Leicester has opened a tuberculosis dispensary. The work of such an institution has been carried on for some months in a necessarily restricted way at the offices of the Health Department at the Town Hall, but now convenient premises, formerly used for business purposes, have been admirably adapted to meet their new requirements. A thorough renovation has been carried out, the walls have been re-plastered and treated with white enamel, and such essential matters as ventilation and light properly attended to. The rooms in use include a waiting-room, dressing-rooms, consulting-room, and doctor's retiring-room. The dispensary will start work with a list of nearly seventy patients, most of whom have been at different times inmates of the local sanatorium. Dr. Killick Millard has secured the cordial co-operation of the medical practitioners of the town, and there is no doubt that the dispensary will achieve a useful work in conjunction with the sanatorium.

### PAYING PATIENTS AT NORTHWOOD.

The Committee of Management of the Mount Vernon Hospital for Consumption has now completed arrangements for admitting paying patients to the sanatorium at Northwood, Middlesex. This new departure will, we believe, prove an acceptable convenience to many consumptives in and around London. Applications should be made to the Secretary at the offices in Fitzroy Square.

### THE DEVON AND EXETER HOSPITAL.

The quarterly meeting of the Governors of the Royal Devon and Exeter Hospital was held last week and was presided over by Mr. Tremayne Buller, president of the hospital. It was announced that a cheque for £1,000 had been received from Mrs. Godfrey Walker for the purpose of endowing two cots in the children's ward, to be named after the donor's husband and sister.

### AN APPEAL AND A CHALLENGE.

It is gratifying to learn that in immediate response to the challenge in favour of King Edward VII.'s Hospital, Cardiff (the Cardiff Infirmary), referred to in our Institutional News columns last week, Lord Merthyr has addressed a letter to Colonel Bruce Vaughan containing the following intimation: "In response to the challenge contained in your letter, I have much pleasure in saying that in order to assist in completing the scheme initiated by you at my request in commemoration of King Edward VII., I shall be glad to contribute £500 with eight other subscribers to make up £4,500 as proposed by 'A Glamorgan Owner of Property.'" His Lordship's letter will afford a special encouragement to the authorities of the Infirmary, following so quickly upon the "challenge," and assuring the continued interest of Lord Merthyr in an institution to which, as Sir William Thomas Lewis, he has already rendered incalculable service. Amongst other responses are gifts of £50 each from Mrs. Jenner, of Wenvoe Castle, a contributor to every appeal that has been issued for the Cardiff Infirmary, and from Mr. David Williams, Llantrisant.

### THIS WEEK'S DRUG MARKET.

Business in the drug market is, generally speaking, rather quiet, but considerable interest continues to be taken in several articles. Prices on the whole are well maintained with, in some instances, an advancing tendency. Menthol has not further advanced in price but, apparently, there is no immediate prospect of any substantial reduction; at the present high figure the demand is quiet and, as mentioned last week, buyers would be wise to limit their purchases to immediate requirements. A good business has been done in rhubarbs at advancing rates, but prices will probably not advance materially higher. The value of eucalyptus oil still tends higher and the present is possibly a suitable time to make purchases to cover the winter needs. Cod-liver oil is in better demand in view of the approaching cold weather, but prices still have a slightly downward tendency. Japanese oil of peppermint is unchanged in value, but the recently advanced price is well maintained. Opium is unchanged, but morphine is rather dearer. The price of alum has been advanced slightly, as also have quotations for ammonia. Cassia oil is dearer. The prices of ergot of rye, hydrastis canadensis, and oil of aniseed are well maintained. Balsam tolu is again dearer and the price of balsam copaiba is tending higher. Chamomiles and oil of cloves are slightly lower in value.

